# Peptidyl Prolyl Isomerase A Modulates the Liquid–Liquid Phase Separation of Proline-Rich IDPs

**DOI:** 10.1021/jacs.2c07149

**Published:** 2022-08-26

**Authors:** Maria Babu, Filippo Favretto, Marija Rankovic, Markus Zweckstetter

**Affiliations:** † Deutsches Zentrum für Neurodegenerative Erkrankungen (DZNE), Von-Siebold Straße 3a, Göttingen, 37075, Germany; ‡ Max Planck Institute for Multidisciplinary Sciences, Am Fassberg 11, Göttingen, 37077, Germany

## Abstract

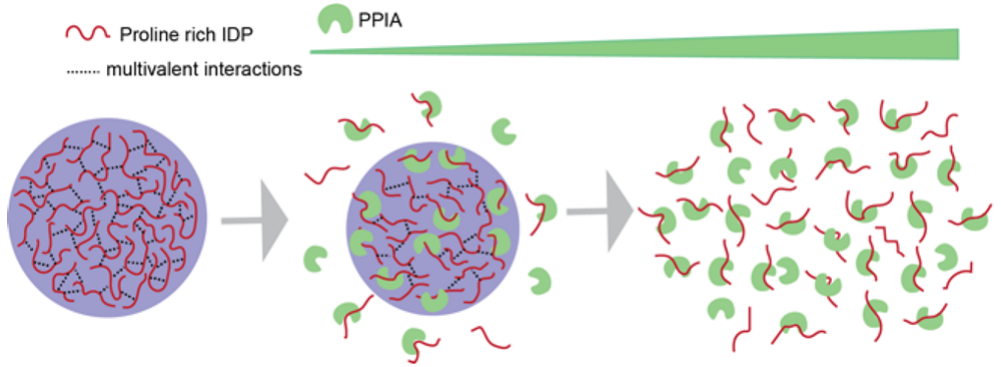

Liquid–liquid phase separation (LLPS) of intrinsically disordered proteins (IDPs) and the action of molecular chaperones are tightly connected. An important class of molecular chaperones are peptidyl prolyl isomerases, which enhance the cis/trans-isomerization of proline. However, little is known about the impact of peptidyl prolyl isomerases on the LLPS of IDPs, which often contain many prolines. Here, we demonstrate that the most ubiquitous peptidyl prolyl isomerase, peptidyl prolyl isomerase A (PPIA), concentrates inside liquid-like droplets formed by the Alzheimer’s disease-associated protein tau, as well as inside RNA-induced coacervates of a proline–arginine dipeptide repeat protein. We further show that the recruitment of PPIA into the IDP droplets triggers their dissolution and return to a single mixed phase. NMR-based binding and proline isomerization studies provide insights into the mechanism of LLPS modulation. Together, the results establish a regulatory role of proline isomerases on the liquid–liquid phase separation of proline-rich IDPs.

## Introduction

Liquid–liquid phase separation (LLPS) of intrinsically disordered proteins/regions (IDPs/IDRs) facilitates the formation of membrane-less organelles,^1^ and aberrant liquid to solid phase transitions are linked to neurotoxicity.^2^ Growing evidence supports an important role of molecular chaperones in regulating LLPS and LLPS-associated biomolecular condensation of IDPs. For example, nuclear-import receptor chaperones can inhibit phase separation of RNA-binding proteins.^3,4^ In addition, the heat shock chaperones HSP70 and HSP27 maintain the liquidity of condensates formed by the amyotrophic lateral sclerosis (ALS)/frontotemporal dementia (FTD)-associated proteins TDP43 and FUS, while a protein disulfide isomerase was shown to repress LLPS and modulate the aggregation of the Alzheimer’s disease-associated protein tau.^5−7^ Molecular chaperones thus might protect proteins from misfolding and pathogenic aggregation inside cellular condensates.

Peptidyl prolyl isomerases are cotranslational chaperones that assist in the folding of nascent amino acid chains.^8−10^ Their chaperoning activity in protein folding is associated with the cis/trans-interconversion of prolines, the only amino acid that can exist in both conformations.^8,11,12^ Apart from their function in protein folding, prolyl isomerases, through the combination of their binding and isomerase activity, are associated with higher order assembly formation of IDPs, particularly the disease-associated misfolding of IDPs into amyloid fibrils: prolyl isomerases such as FK506-binding proteins, cyclophilin A, and cyclophilin D modulate the amyloid fibril formation of proline-rich IDPs associated with neurodegeneration including tau and α-synuclein.^13−17^ In contrast to the regulatory activity of prolyl isomerases on the fibril formation of IDPs, their regulatory role on the LLPS behavior of IDPs is unknown.

LLPS is a metastable protein assembly often mediated by IDPs.^18^ Prolines are about 1.7–1.8 times more abundant in IDPs when compared to structured proteins.^19^ Thus, the effect of prolyl isomerases, with their unique action on proline residues, is intriguing in the context of LLPS. The peptidyl prolyl isomerase A (PPIA) is the most abundant prolyl isomerase in cells.^20^ Consistent with an important role of prolyl isomerases in LLPS regulation, a recent study found that the interactome of PPIA is enriched in proline-rich IDR-containing DNA/RNA binding proteins involved in biomolecular condensation.^10^ In addition, PPIA localizes within stress granules,^21^ a biomolecular condensate formed by DNA/RNA binding proteins in cellular stress conditions or disease-associated conditions.^22,23^ The expression of PPIA is also known to vary during cellular stress.^24,25^ These observations suggest a broad biological significance of the prolyl isomerase PPIA in the regulation of biomolecular condensation.

Here, we provide molecular insights into the enigmatic role of PPIA in regulating the LLPS behavior of proline-rich IDPs. In order to provide insight into the influence of PPIA on both self-coacervation and complex coacervation, we studied the Alzheimer’s disease-associated protein tau, which can undergo LLPS without nucleic acids,^26,27^ as well as the 40-residue proline–arginine dipeptide repeat protein PR20, which most efficiently phase separates with RNA.^28,29^ The transition of tau from liquid condensates to a solid phase is associated with tau aggregation into insoluble deposits.^30^ PR dipeptide repeat proteins are abnormally expressed in the brain of patients with C9-ALS/FTD.^31^ The toxicity of PR dipeptide repeat proteins in C9-ALS/FTD is linked to their incorporation into membrane-less compartments, thus changing their properties.^28,32^ Previous studies demonstrated that the dipeptide repeat protein PR20 interacts with PPIA *in vitro* and in cells.^32,33^

## Results

**Figure 1:**
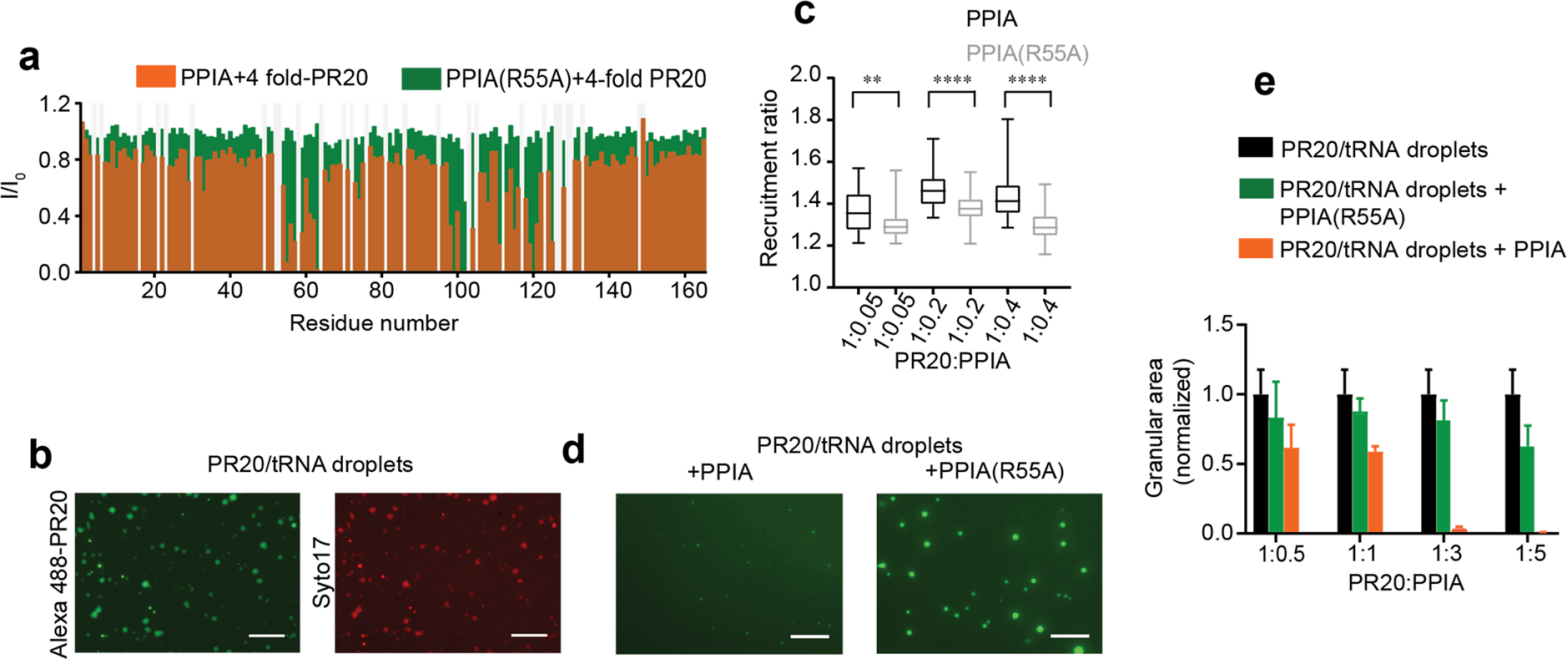
PPIA interferes with RNA-induced LLPS of PR20. (a) Single-residue analysis of the interaction of PR20 with wild-type and mutant PPIA. Changes in the intensities of ^1^H–^15^N HSQC peaks of PPIA (orange) and PPIA(R55A) (green) upon addition of a 4-fold excess of PR20. *I* and *I*_0_ are the intensities of the PPIA HSQC peaks in the presence and absence of PR20, respectively. Light gray bars represent residues that are excluded from the analysis. (b) LLPS of PR20 into PR20/tRNA droplets. Droplets were visualized by addition of Alexa488-labeled PR20 (green) and Syto 17 RNA dye (red). Images were obtained after 15 min of incubation. Scale bar, 20 μm. (c) Concentration of PPIA inside PR20/tRNA droplets. Recruitment ratios calculated on the basis of ∼30 droplets for each PR20:PPIA (black) and PR20:PPIA(R55A) (gray) molar ratio. In the box and whisker plot, the middle line is the median, ends of boxes represent the upper and lower quartiles, while whiskers extend until the highest and lowest observations. The difference within a PR20:PPIA ratio was analyzed by an unpaired *t* test: **p < 0.0017, ****p < 0.0001. For *p* < 0.05, the two data sets are considered to be significantly different. (d) PPIA-induced dissolution of PR20/tRNA droplets. Fluorescence images of Alexa488-labeled PR20/tRNA droplets with PPIA (left) and PPIA(R55A) (right) at a PR20:PPIA (or PPIA(R55A)) molar ratio of 1:3. Images were obtained after 15 min of incubation. Scale bar, 20 μm. (e) Granular areas occupied by PR20/tRNA droplets 15 min after addition of wild-type PPIA (orange) or mutant PPIA(R55A) (green) for PR20:PPIA (or PPIA(R55A) ratios of 1:0.5, 1:1, 1:3, and 1:5. Granular areas in a control sample without PPIA are displayed in black. Granular area is taken as the average of area occupied by droplets in four micrographs. Error bars represent standard deviation from average.

To gain single-residue resolution insight into the interaction of PPIA with the dipeptide repeat protein PR20, we used NMR spectroscopy. In agreement with previous results,^33^ PR20 induced strong signal broadening of selected PPIA residues. The cross peak of Arg55, the PPIA residue that is crucial for its catalytic activity,^34^ was broadened beyond detection (Figure S1a). In addition, several other residues in the active site of PPIA were perturbed (Figures 1a, S1a). In the crystal structure of the PPIA/PR20 complex, Arg55 forms a hydrogen bond with the carbonyl group of a proline–arginine peptide of PR20.^33^ The binding affinity of this interaction, as determined from the intensity perturbations of Arg55 and Asn102, is 23 μM (Figure S1c). Next, we repeated the experiments with the mutant protein PPIA(R55A), in which Arg55 is mutated to alanine.^35^ This mutation was previously shown to attenuate its binding to substrates and to decrease its cis/trans-isomerization activity. In contrast to wild-type PPIA, only little signal broadening was observed in the ^1^H–^15^N correlation spectrum of PPIA(R55A) upon addition of PR20 (Figures 1a, S1b). Only a few residues in the active site experienced residual chemical shift perturbations, in particular Asn102 (Figure S1b), which is in contact with an arginine side chain of PR20 in the PPIA/PR20 complex.^33^ Comparison of the intensity perturbations of Arg55 and its mutant Ala55 at increasing PR20 concentrations highlights the difference in affinity of PR20 to wild-type PPIA and the mutant PPIA(R55A) (Figure S1c).

Next, we investigated the effect of PPIA on the complex coacervation of PR20 with RNA. Previous studies showed that PR20 efficiently forms liquid-like droplets upon addition of tRNA.^28,29^ Consistent with these studies, we observed LLPS of 100 μM PR20 when mixing it with 0.2 mg/mL tRNA (Figures 1b, S2a). Using fluorescence microscopy, PR20/tRNA droplets were observable for ∼1–1.5 h after mixing the two components (Figure S2d). A similar time-dependent instability of peptide–RNA coacervates was previously reported.^36^ The effect of PPIA on PR20/tRNA droplets was therefore studied during this time window.

We then quantified the degree of PPIA recruitment into PR20/tRNA droplets. This was achieved by calculating the ratio of fluorescence intensity of PPIA inside and outside of similar-sized droplets. For different PR20:PPIA molar ratios (1:0.05, 1:0.2, 1:0.4, i.e., a large excess of PR20 over PPIA), PPIA concentrated inside the PR20/tRNA droplets (Figures 1c, S2b). We also repeated the experiments with the mutant PPIA(R55A). Fluorescence microscopy showed that PPIA(R55A) concentrates inside of PR20/tRNA droplets. Its recruitment was slightly attenuated when compared to wild-type PPIA (Figure 1c), likely due to the lower affinity to PR20 (Figure S1c).

We then investigated the effect of higher concentrations of PPIA and PPIA(R55A) on PR20/tRNA droplets. PPIA (PPIA(R55A)) was added to the droplets at PR20:PPIA molar ratios of 1:0.5, 1:1, 1:3, and 1:5 (Figures 1d,e, S2c,d). The mutant PPIA did not dissolve the PR20/tRNA droplets at the tested concentrations (Figures 1d,e, S2c,d). At 5-fold excess of PPIA(R55A) over PR20, the amount of droplets was slightly decreased (Figure 1e), potentially due to residual binding (Figure S1b). In contrast, we observed immediate complete dissolution of the PR20/tRNA droplets upon addition of a 3- or 5-fold molar excess of wild-type PPIA. At equimolar concentrations of wild-type PPIA and PR20, PR20-LLPS was partially diminished (Figures 1d,e, S2d). We attribute the finding that at equimolar concentration the droplets are not fully dissolved to a combination of factors, including the incomplete recruitment of PPIA to the droplets (Figure 1c) and the competition between PPIA and RNA for binding to PR20.

Next, we probed the effect of PPIA on the dynamics of PR20 inside the PR20/tRNA droplets. PPIA (or PPIA(R55A)) was added to PR20/tRNA droplets at a substoichiometric PR20:PPIA (1:0.4) molar ratio. We photobleached fluorescently labeled PR20 inside the droplets and recorded the recovery rate (Figure S3a). The rate of fluorescence recovery was similar in the presence of either PPIA or PPIA(R55A) (Figure S3b). In addition, the recovery rate was comparable to that in a reference sample where neither variant was present. This showed that PPIA did not affect the dynamics inside PR20/tRNA droplets at this low PPIA concentration.

**Figure 2:**
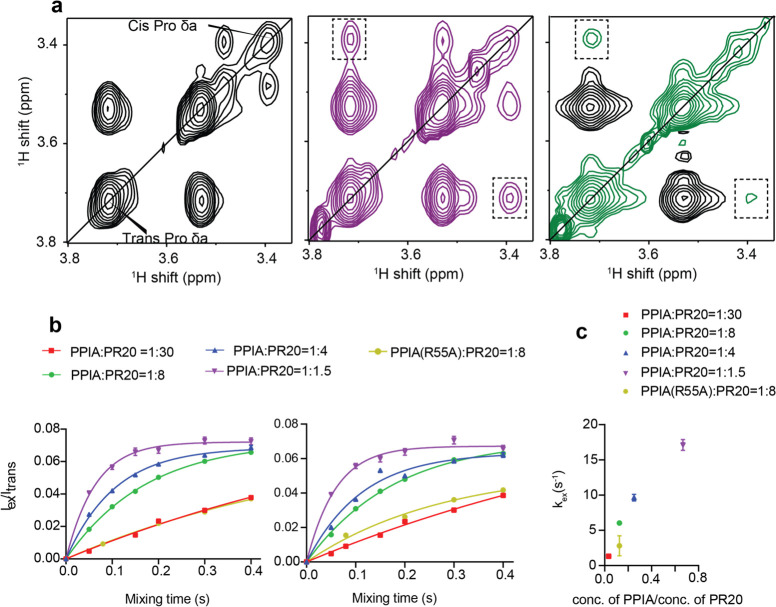
Isomerase activity of PPIA on the dipeptide repeat protein PR20 in the dilute state in the absence of RNA. (a) NOESY spectrum of PR20 alone (left) and in the presence of PPIA (middle; PPIA:PR20 molar ratio of 1:8) in the region of H^δ^ of prolines. The exchange peak between the cis and trans isoforms of H^δ^ proline is marked by a rectangle. For comparison, the ROESY spectrum of the same PPIA/PR20 sample is shown on the right. The mixing time for the NOESY experiments is 300 ms; for the ROESY it is 220 ms. (b) Ratios between the intensity of the cis/trans-exchange peak of proline H^δ^, *I*(ex), and the intensity of its trans diagonal peak, *I*(trans), as a function of mixing time of the NOESY experiment for PPIA:PR20 molar ratios of 1:30 (red, square), 1:8 (green, circle),1:4 (blue, triangle), and 1:1.5 (magenta, inverted triangle) and for a PPIA(R55A):PR20 ratio of 1:8 (yellow, circle). Lines represent least-squares fittings of the data to obtain the exchange rate *k*_ex_. Error bars represent the error in *I*_ex_/*I*_trans_ calculated from the noise in the NMR spectra. The graphs on the left and right represent the same analysis, but the *I*_ex_ value in the two cases is taken from the two exchange peaks on either side of the diagonal, which are marked by rectangular boxes in panel a (middle). (c) Rates of cis/trans-interconversion, *k*_ex_, in PR20 for different PPIA:PR20 ratios derived from fitting the *I*_ex_/*I*_trans_ value corresponding to various mixing times against eq 7. Error bars represent standard deviations from the average *k*_ex_ value.

To investigate the ability of PPIA to catalyze the cis/trans-isomerization of the proline residues of PR20, we utilized NOESY and ROESY NMR spectroscopy. NOESY and ROESY experiments are powerful methods to probe two-state exchange processes within the range of the NOE/ROE mixing time (10^–3^ s) including proline isomerization.^37^ In the two-dimensional NOESY spectrum of PR20 in the dilute state in the absence of RNA, all prolines and all the arginine residues have overlapping chemical shifts because of the repeat nature of the peptide. The NOESY spectrum of PR20 recorded in the presence of PPIA, when compared to the same for PR20 alone, displayed an additional exchange cross peak between the cis and trans isoforms of H^δ^ of proline (Figure 2a). This exchange peak suggests that the cis/trans-exchange of arginine–proline peptide bonds in PR20 shifted in the presence of PPIA to a faster time scale, which is detectable within the NOESY observation time. To verify that the additional cross peak is an exchange peak, a ROESY spectrum was recorded. In the ROESY spectrum, the same sign of the additional peak with respect to the diagonal peak confirmed an exchange process as the source of this cross peak (Figure 2a).

To quantify the enhancement in isomerization rate in PR20 in the presence of PPIA, the cis/trans-interconversion rate (*k*_ex_ value) was determined for PPIA:PR20 molar ratios of 1:30, 1:8, 1:4, and 1:1.5. Experimental data, i.e., the ratio of intensity of the exchange peak to that of the trans diagonal peak (or the cis diagonal peak), derived from NOESY spectra with mixing times ranging from 50 to 400 ms were fitted according to the two-state exchange model for proline isomerization (Figures 2b, S4a). The cis/trans-interconversion rates for proline in a peptide are on the order of 10^–3^ s^–1^ in the absence of isomerases.^38^ The *k*_ex_ value estimated for prolines of PR20 in the presence of PPIA was higher than this value by about 3 orders of magnitude. The average *k*_ex_ values derived from the intensity ratio of the exchange peak to the trans diagonal peak are 1.33 ± 0.01 s^–1^, 6.05 ± 0.11 s^–1^, 9.64 ± 0.46 s^–1^, and 17.13 ± 0.77 s^–1^ for PPIA:PR20 molar ratios of 1:30, 1:8, 1:4, and 1:1.5, respectively (Figure 2c). When derived from the intensity ratios of the exchange peak to the cis diagonal peak, we obtained 2.99 ± 0.20 s^–1^, 8.45 ± 0.08 s^–1^, 14.86 ± 1.03 s^–1^, and 20.80 ± 7.36 s^–1^, respectively (Figure S4b). The later *k*_ex_ values are less accurate, because of the low signal intensity of the cis diagonal peak (Figure 2a). We attribute the differences in the *k*_ex_ values derived from the two ways of analysis to the inaccuracies in the later “cis” analysis. Notably, the interconversion rate gradually increases with increasing PPIA concentration and the dependence of *k*_ex_ on PPIA concentration starts to saturate at higher PPIA concentrations (PPIA:PR20 of 1:1.5) (Figures 2c, S4b).

Following the same strategy, we then determined the *k*_ex_ value of proline isomerization in PR20 in the presence of the mutant PPIA(R55A) (Figures 2b,c, S4a,b). At an 8-fold excess of PR20 over PPIA(R55A), the *k*_ex_ value obtained from the intensity ratio of the exchange peak to the trans diagonal peak was 2.82 ± 1.42 s^–1^, and that from the intensity ratio of the exchange peak to the cis diagonal peak 4.56 ± 2.03. The *k*_ex_ values in the presence of the mutant PPIA(R55A) are thus approximately a factor 2 lower than with the wild-type PPIA (Figures 2c, S4b). This demonstrates the residual activity of PPIA(R55A). A complete inhibition of the enzymatic activity would require a full blockage of the binding, underlining the difficulty of disentangling the effect of binding and isomerization on droplet dissolution.

**Figure 3:**
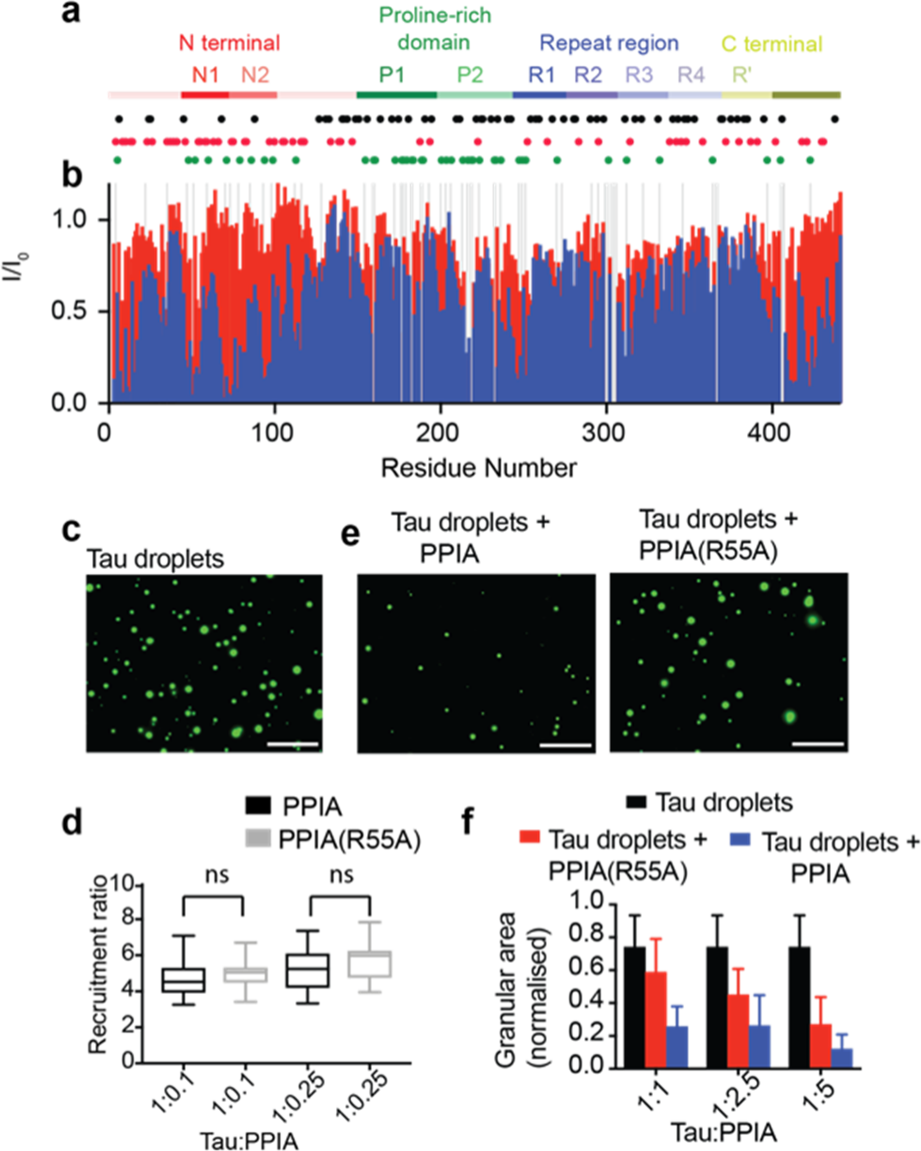
PPIA modulates tau LLPS. (a) Domain organization of tau comprising the N-terminal domain, the proline-rich domain (P1 and P2), the repeats R1, R2, R3, R4, and R′, and the C-terminal domain. The locations of prolines are marked by green dots. Black and red dots represent positively and negatively charged residues, respectively. (b) Binding of tau to wild-type and mutant PPIA. Changes in the intensities of ^1^H–^15^N HSQC peaks of tau upon addition of a 10-fold excess of PPIA (blue) and PPIA(R55A) (red). *I* and *I*_0_ are the intensities of the tau HSQC peaks in the presence and absence of PPIA (or PPIA(R55A)), respectively. Gray bars represent residues that are excluded from the analysis. (c) LLPS of tau. Droplets were visualized by addition of Alexa488-labeled tau (green). Image was recorded after 5 min of incubation. Scale bar, 30 μm. (d) Concentration of PPIA inside tau droplets. Recruitment ratios calculated on the basis of ∼20 droplets for each tau:PPIA (black) and tau:PPIA(R55A) (gray) molar ratio, obtained from two independent experiments per condition. In the box and whisker plot, the middle line is the median, ends of the boxes represent the upper and lower quartiles, while whiskers extend until the highest and lowest observations. An unpaired *t* test gave no significant difference between PPIA and PPIA(R55A) recruitment ratios. ns stands for no significant difference, i.e., *p* > 0.05. (e) PPIA-induced dissolution of tau droplets. Fluorescence images of Alexa488-labeled tau droplets with PPIA (left) and PPIA(R55A) (right) at a PR20:PPIA (or PPIA(R55A)) ratio of 1:1. Images were obtained after 5 min of incubation. Scale bar, 30 μm. (f) Granular areas occupied by tau droplets 5 min after addition of wild-type PPIA (blue) or mutant PPIA(R55A) (red) for PR20:PPIA (or PPIA(R55A)) ratios of 1:1, 1:2.5, and 1:5. Granular areas in a control sample without PPIA are displayed in black. Granular area is taken as the average of area occupied by droplets, calculated from 24 images from three repeats (8 images from one repeat) per condition. Error bars represent standard deviation from average area.

Next, we investigated if PPIA is able to reverse LLPS of a proline-rich IDP, which does not require nucleic acids for LLPS. We selected the 441 residue protein tau (Figure 3a), because it has—in addition to its importance for disease—several useful properties: (i) a more diverse amino acid sequence when compared to PR20, (ii) a high content of proline residues in the so-called proline-rich region (Figure 3a), which is important for tau LLPS,^39^ and (iii) robust self-coacervation at room temperature.^36^ First, we characterized the binding of PPIA to tau using NMR (Figures 3b, S5). Residue-specific analysis showed that PPIA decreases the signal intensity of many tau cross peaks in the 2D ^1^H–^15^N HSQC. The strongest signal attenuation was detected at the N-terminus of tau, in and close to the two N-terminal inserts N1/N2, the proline-rich domain, repeats R1 and R3, and the C-terminal region (Figure 3b). Much less signal broadening was induced in the tau cross peaks when the mutant PPIA(R55A) was added (Figure 3b, red).

In order to gain further insights into the PPIA/tau interaction, we titrated ^15^N-labeled PPIA with unlabeled tau. Only at very high molar excess of tau over PPIA did we detect changes in the position and intensity of the PPIA cross peaks (Figure S6a,c). This is in strong contrast to the NMR data for the PPIA/tau titration, in which strong signal broadening already occurred at 4-fold excess of PR20 over PPIA (Figure 1a). We then performed a residue-specific analysis of the tau-induced chemical shift perturbations in PPIA (Figure S6c). The analysis showed that the tau-induced changes were located in PPIA’s enzymatic pocket (Figure S6d). Fitting the concentration-dependent chemical shift perturbation (CSP) of Arg55 to a one-site binding model results in a *K*_d_ value of 353 ± 30 μM (Figure S6e). We then performed a global fit of the CSPs of several strongly perturbed residues (Arg55, Met61, Ser99, Phe113, Thr119, Leu122) and obtained a *K*_d_ value of 194 ± 39 μM. The affinity of the PPIA/tau interaction is thus approximately a magnitude weaker than the PPIA/PR20 interaction. On the basis of the calculated *K*_d_ values, we estimate that at the conditions of the NMR experiment shown in Figure 3b ∼49% (global fit; 35% for the R55-fit) of tau molecules are bound to PPIA. Because the degree of PPIA-induced signal broadening largely exceeds those values in several tau regions (Figure 3b), we conclude that a sizeable fraction of the signal broadening induced in tau upon PPIA addition likely arises from PPIA-catalyzed cis/trans-isomerization of tau’s proline residues.

We also titrated ^15^N-labeled mutant PPIA(R55A) with tau. We observed chemical shift perturbations that were weaker than those of the wild-type PPIA/tau interaction (Figure S6b,c), while the signal broadening was comparable (Figure S6c). Estimation of the *K*_d_ on the basis of the chemical shift perturbation returned values of 817 ± 74 μM (for Arg55; Figure S6e) and 562 ± 83 μM (for global fit). Thus, the PPIA-bound fraction of tau molecules in the NMR experiment of Figure 3b is 19% (on the basis of the Arg55 *K*_d_) and 26% (for the global fit *K*_d_).

Next, we studied the impact of both wild-type and mutant PPIA on tau LLPS. Tau undergoes LLPS at 20 μM concentration in a buffer of low ionic strength (Figures 3c, S7a).^36^ When PPIA is added, it is enriched 4–6-fold inside the tau droplets (Figure 3d). A similar enrichment was observed for the mutant PPIA(R55A) (Figures 3d, S7b). The more pronounced enrichment of PPIA inside tau droplets (4–6-fold) when compared to PR20/tRNA droplets (∼1.4) suggests that in the case of PR20/tRNA the competitive binding between PPIA and tRNA to PR20 decreases the enrichment of PPIA inside the PR20/tRNA droplets. In subsequent experiments, we added PPIA to preformed tau droplets at 1:1, 1:2.5, and 1:5 molar ratios (Figures 3e,f, S7c). This caused a strong decrease in tau droplet numbers already at equimolar concentration (Figures 3e,f, S6c). In the case of the mutant PPIA(R55A), less dissolution was detected (Figures 3e,f, S7c). We further note that lower concentrations of PPIA are required to dissolve tau droplets than PR20/tRNA droplets, despite the reduced affinity of PPIA to tau.

Next, PPIA was added to tau droplets at a tau:PPIA molar ratio of 1:0.5. Fluorescently labeled tau inside a region of the droplet was photobleached, and the recovery was recorded (Figure S8a). The recovery rate was comparable for tau droplets in the presence and absence of PPIA (Figure S8b). Thus, for both droplet systems, tau and PR20/RNA, recruitment of PPIA did not cause a detectable change of the liquidity of the protein/polypeptide inside the droplets.

## Discussion

Different chaperones have been investigated with respect to their regulatory role in biomolecular LLPS,^3−6,40−42^ but the role of PPIA or other prolyl isomerases, despite the abundance of phase separating proteins in its interactome,^10^ remained unexplored. Using the proline-rich proteins tau and PR20, we showed that PPIA is recruited into and dissolves liquid-like droplets formed by these IDPs. PPIAs are special when compared to other chaperones for two reasons: (i) they preferentially bind to proline residues, and (ii) they catalyze proline cis/trans-isomerization. Generally, it is difficult to decouple these two processes, because both occur at the active site; that is, point mutations affect both processes. Despite the strong connection between binding to the active site and catalysis of proline isomerization, the modulatory action of PPIA on tau LLPS points to a significant contribution of proline isomerization to the PPIA-mediated dissolution of tau droplets (Figure 3). Binding of PPIA to tau is very weak such that in both the dilute phase and, even more, inside the droplets, where tau is highly concentrated, only a small fraction of tau molecules are bound to PPIA. When we make some simplifying assumptions such as (i) all tau is inside the droplet (in agreement with negligible tau fluorescence outside; Figure 3c,e), (ii) the area occupied by the droplets is directly correlated to the volume, i.e., the third dimension of the slice observed under the microscope is considered negligible, and (iii) one-site binding of tau to PPIA, we estimated the fraction of PPIA-bound tau inside the droplets as ∼1.5% at the tau:PPIA molar ratio of 1:0.25 (3.4% at the tau:PPIA molar ratio of 1:0.5). Because the recruitment of wild-type PPIA and mutant PPIA(R55A) into the droplets is very similar (Figure 3d) and the affinity of PPIA(R55A) is only ∼2–3-fold lower (Figure S6e), this value changes only to ∼1.2% at the tau:PPIA(R55A) molar ratio of 1:0.25 (2.7% at the tau:PPIA(R55A) molar ratio of 1:0.5). In contrast, we find that PPIA drastically remodels the conformational ensemble of tau as seen by PPIA-induced signal broadening of the tau backbone resonances (Figure 3b). This remodeling is largely absent for the mutant PPIA (Figure 3b). We thus suggest that the stronger dissolution power of PPIA when compared to PPIA(R55A) (Figure 3e,f) is linked to the wild-type protein’s ability to remodel the conformational ensemble of tau through proline isomerization.

In the current study we have investigated the regulatory role of the proline isomerase PPIA on liquid-like droplets freshly formed by two proline-rich IDPs. Changes in the material properties of droplets from a liquid-like state to more solid phases, however, have been linked to amyloid formation, for example in the case of the ALS/FTD-related protein FUS and also for tau.^30,43^ It will therefore be interesting to study how PPIA and other proline isomerases modulate the maturation kinetics of condensates. Because of the strong changes induced in the conformational ensembles of IDPs by proline isomerization, the maturation kinetics of condensates could be affected by proline isomerases. Supportive for this hypothesis are studies in cells: PPIA expression was essential for stress granule formation in hematopoietic cells in conditions of oxidative stress,^10^ and knock out or age-dependent reduction of PPIA decreased stress granules.^10^

In summary, our work establishes a regulatory role of proline isomerases on the liquid–liquid phase separation of proline-rich IDPs. Targeting proline isomerases by small molecules might thus provide a viable strategy to modulate disease-associated biomolecular condensates.

## Experimental Section

Detailed experimental methods are included in the Supporting Information.

## Supporting Information Available

Experimental details and supporting figures (PDF)

## References

[ref1] Banani S. F.; Lee H. O.; Hyman A. A.; Rosen M. K. Biomolecular condensates: organizers of cellular biochemistry. Nat. Rev. Mol. Cell Biol. 2017, 18 (5), 285–298. 10.1038/nrm.2017.7. 28225081 PMC7434221

[ref2] Spannl S.; Tereshchenko M.; Mastromarco G. J.; Ihn S. J.; Lee H. O. Biomolecular condensates in neurodegeneration and cancer. Traffic 2019, 20 (12), 890–911. 10.1111/tra.12704. 31606941

[ref3] Guo L.; Kim H. J.; Wang H.; Monaghan J.; Freyermuth F.; Sung J. C.; O’Donovan K.; Fare C. M.; Diaz Z.; Singh N.; Zhang Z. C.; Coughlin M.; Sweeny E. A.; DeSantis M. E.; Jackrel M. E.; Rodell C. B.; Burdick J. A.; King O. D.; Gitler A. D.; Lagier-Tourenne C.; Pandey U. B.; Chook Y. M.; Taylor J. P.; Shorter J. Nuclear-Import Receptors Reverse Aberrant Phase Transitions of RNA-Binding Proteins with Prion-like Domains. Cell 2018, 173 (3), 677–692. 10.1016/j.cell.2018.03.002. 29677512 PMC5911940

[ref4] Hofweber M.; Hutten S.; Bourgeois B.; Spreitzer E.; Niedner-Boblenz A.; Schifferer M.; Ruepp M. D.; Simons M.; Niessing D.; Madl T.; Dormann D. Phase Separation of FUS Is Suppressed by Its Nuclear Import Receptor and Arginine Methylation. Cell 2018, 173 (3), 706–719. 10.1016/j.cell.2018.03.004. 29677514

[ref5] Yu H.; Lu S.; Gasior K.; Singh D.; Vazquez-Sanchez S.; Tapia O.; Toprani D.; Beccari M. S.; Yates J. R. 3rd; Da Cruz S.; Newby J. M.; Lafarga M.; Gladfelter A. S.; Villa E.; Cleveland D. W. HSP70 chaperones RNA-free TDP-43 into anisotropic intranuclear liquid spherical shells. Science 2021, 371 (6529), eabb4309 10.1126/science.abb4309. 33335017 PMC8286096

[ref6] Liu Z.; Zhang S.; Gu J.; Tong Y.; Li Y.; Gui X.; Long H.; Wang C.; Zhao C.; Lu J.; He L.; Li Y.; Liu Z.; Li D.; Liu C. Hsp27 chaperones FUS phase separation under the modulation of stress-induced phosphorylation. Nat. Struct Mol. Biol. 2020, 27 (4), 363–372. 10.1038/s41594-020-0399-3. 32231288

[ref7] Wang K.; Liu J. Q.; Zhong T.; Liu X. L.; Zeng Y.; Qiao X.; Xie T.; Chen Y.; Gao Y. Y.; Tang B.; Li J.; Zhou J.; Pang D. W.; Chen J.; Chen C.; Liang Y. Phase Separation and Cytotoxicity of Tau are Modulated by Protein Disulfide Isomerase and S-nitrosylation of this Molecular Chaperone. J. Mol. Biol. 2020, 432 (7), 2141–2163. 10.1016/j.jmb.2020.02.013. 32087196

[ref8] Schmid F. X. Prolyl isomerase: enzymatic catalysis of slow protein-folding reactions. Annu. Rev. Biophys. Biomol. Struct. 1993, 22, 123–42. 10.1146/annurev.bb.22.060193.001011. 7688608

[ref9] Gothel S. F.; Marahiel M. A. Peptidyl-prolyl cis-trans isomerases, a superfamily of ubiquitous folding catalysts. Cell. Mol. Life Sci. 1999, 55 (3), 423–36. 10.1007/s000180050299. 10228556 PMC11146858

[ref10] Maneix L.; Iakova P.; Moree S. E.; King J. C. K.; Sykes D. B.; Hill C. T.; Saez B.; Spooner E.; Krause D. S.; Sahin E. Cyclophilin A regulates protein phase separation and mitigates haematopoietic stem cell aging. bioRxiv. 2021, 10.1101/2021.02.24.432737 (accessed 2022–08–19).

[ref11] Schmid F. X.; Mayr L. M.; Mucke M.; Schonbrunner E. R. Prolyl isomerases: role in protein folding. Adv. Protein Chem. 1993, 44, 25–66. 10.1016/S0065-3233(08)60563-X. 8317297

[ref12] Lang K.; Schmid F. X.; Fischer G. Catalysis of protein folding by prolyl isomerase. Nature 1987, 329 (6136), 268–70. 10.1038/329268a0. 3306408

[ref13] Favretto F.; Flores D.; Baker J. D.; Strohaker T.; Andreas L. B.; Blair L. J.; Becker S.; Zweckstetter M. Catalysis of proline isomerization and molecular chaperone activity in a tug-of-war. Nat. Commun. 2020, 11 (1), 6046 10.1038/s41467-020-19844-0. 33247146 PMC7695863

[ref14] Baker J. D.; Shelton L. B.; Zheng D.; Favretto F.; Nordhues B. A.; Darling A.; Sullivan L. E.; Sun Z.; Solanki P. K.; Martin M. D.; Suntharalingam A.; Sabbagh J. J.; Becker S.; Mandelkow E.; Uversky V. N.; Zweckstetter M.; Dickey C. A.; Koren J. 3rd; Blair L. J. Human cyclophilin 40 unravels neurotoxic amyloids. PLoS Biol. 2017, 15 (6), e2001336 10.1371/journal.pbio.2001336. 28654636 PMC5486962

[ref15] Torpey J.; Madine J.; Wood A.; Lian L. Y. Cyclophilin D binds to the acidic C-terminus region of alpha-Synuclein and affects its aggregation characteristics. Sci. Rep 2020, 10 (1), 10159 10.1038/s41598-020-66200-9. 32576835 PMC7311461

[ref16] Gerard M.; Deleersnijder A.; Daniels V.; Schreurs S.; Munck S.; Reumers V.; Pottel H.; Engelborghs Y.; Van den Haute C.; Taymans J. M.; Debyser Z.; Baekelandt V. Inhibition of FK506 binding proteins reduces alpha-synuclein aggregation and Parkinson’s disease-like pathology. J. Neurosci. 2010, 30 (7), 2454–63. 10.1523/JNEUROSCI.5983-09.2010. 20164329 PMC6634531

[ref17] Gerard M.; Debyser Z.; Desender L.; Kahle P. J.; Baert J.; Baekelandt V.; Engelborghs Y. The aggregation of alpha-synuclein is stimulated by FK506 binding proteins as shown by fluorescence correlation spectroscopy. FASEB J. 2006, 20 (3), 524–6. 10.1096/fj.05-5126fje. 16410343

[ref18] Alberti S. Phase separation in biology. Curr. Biol. 2017, 27 (20), R1097–R1102. 10.1016/j.cub.2017.08.069. 29065286

[ref19] Theillet F. X.; Kalmar L.; Tompa P.; Han K. H.; Selenko P.; Dunker A. K.; Daughdrill G. W.; Uversky V. N. The alphabet of intrinsic disorder: I. Act like a Pro: On the abundance and roles of proline residues in intrinsically disordered proteins. Intrinsically Disord Proteins 2013, 1 (1), e24360 10.4161/idp.24360. 28516008 PMC5424786

[ref20] Nigro P.; Pompilio G.; Capogrossi M. C. Cyclophilin A: a key player for human disease. Cell Death Dis 2013, 4, e888 10.1038/cddis.2013.410. 24176846 PMC3920964

[ref21] Xiang S.; Kato M.; Wu L. C.; Lin Y.; Ding M.; Zhang Y.; Yu Y.; McKnight S. L. The LC Domain of hnRNPA2 Adopts Similar Conformations in Hydrogel Polymers, Liquid-like Droplets, and Nuclei. Cell 2015, 163 (4), 829–39. 10.1016/j.cell.2015.10.040. 26544936 PMC4879888

[ref22] Wolozin B.; Ivanov P. Stress granules and neurodegeneration. Nat. Rev. Neurosci 2019, 20 (11), 649–666. 10.1038/s41583-019-0222-5. 31582840 PMC6986315

[ref23] Wolozin B. Regulated protein aggregation: stress granules and neurodegeneration. Mol. Neurodegener 2012, 7, 56 10.1186/1750-1326-7-56. 23164372 PMC3519755

[ref24] Andreeva L.; Heads R.; Green C. J. Cyclophilins and their possible role in the stress response. Int. J. Exp Pathol 1999, 80 (6), 305–15. 10.1046/j.1365-2613.1999.00128.x. 10632780 PMC2517841

[ref25] Santos A. N.; Korber S.; Kullertz G.; Fischer G.; Fischer B. Oxygen stress increases prolyl cis/trans isomerase activity and expression of cyclophilin 18 in rabbit blastocysts. Biol. Reprod. 2000, 62 (1), 1–7. 10.1095/biolreprod62.1.1. 10611060

[ref26] Drubin D. G.; Kirschner M. W. Tau protein function in living cells. J. Cell Biol. 1986, 103, 2739–46. 10.1083/jcb.103.6.2739. 3098742 PMC2114585

[ref27] Kadavath H.; Hofele R. V.; Biernat J.; Kumar S.; Tepper K.; Urlaub H.; Mandelkow E.; Zweckstetter M. Tau stabilizes microtubules by binding at the interface between tubulin heterodimers. Proc. Natl. Acad. Sci. U. S. A. 2015, 112 (24), 7501–6. 10.1073/pnas.1504081112. 26034266 PMC4475932

[ref28] Boeynaems S.; Bogaert E.; Kovacs D.; Konijnenberg A.; Timmerman E.; Volkov A.; Guharoy M.; De Decker M.; Jaspers T.; Ryan V. H.; Janke A. M.; Baatsen P.; Vercruysse T.; Kolaitis R. M.; Daelemans D.; Taylor J. P.; Kedersha N.; Anderson P.; Impens F.; Sobott F.; Schymkowitz J.; Rousseau F.; Fawzi N. L.; Robberecht W.; Van Damme P.; Tompa P.; Van Den Bosch L. Phase Separation of C9orf72 Dipeptide Repeats Perturbs Stress Granule Dynamics. Mol. Cell 2017, 65 (6), 1044–1055. 10.1016/j.molcel.2017.02.013. 28306503 PMC5364369

[ref29] Boeynaems S.; Holehouse A. S.; Weinhardt V.; Kovacs D.; Van Lindt J.; Larabell C.; Van Den Bosch L.; Das R.; Tompa P. S.; Pappu R. V.; Gitler A. D. Spontaneous driving forces give rise to protein-RNA condensates with coexisting phases and complex material properties. Proc. Natl. Acad. Sci. U. S. A. 2019, 116 (16), 7889–7898. 10.1073/pnas.1821038116. 30926670 PMC6475405

[ref30] Wegmann S.; Eftekharzadeh B.; Tepper K.; Zoltowska K. M.; Bennett R. E.; Dujardin S.; Laskowski P. R.; MacKenzie D.; Kamath T.; Commins C.; Vanderburg C.; Roe A. D.; Fan Z.; Molliex A. M.; Hernandez-Vega A.; Muller D.; Hyman A. A.; Mandelkow E.; Taylor J. P.; Hyman B. T. Tau protein liquid-liquid phase separation can initiate tau aggregation. EMBO J. 2018, 37 (7), e98049 10.15252/embj.201798049. 29472250 PMC5881631

[ref31] Freibaum B. D.; Taylor J. P. The Role of Dipeptide Repeats in C9ORF72-Related ALS-FTD. Front Mol. Neurosci 2017, 10, 35 10.3389/fnmol.2017.00035. 28243191 PMC5303742

[ref32] Lee K. H.; Zhang P.; Kim H. J.; Mitrea D. M.; Sarkar M.; Freibaum B. D.; Cika J.; Coughlin M.; Messing J.; Molliex A.; Maxwell B. A.; Kim N. C.; Temirov J.; Moore J.; Kolaitis R. M.; Shaw T. I.; Bai B.; Peng J.; Kriwacki R. W.; Taylor J. P. C9orf72 Dipeptide Repeats Impair the Assembly, Dynamics, and Function of Membrane-Less Organelles. Cell 2016, 167 (3), 774–788. 10.1016/j.cell.2016.10.002. 27768896 PMC5079111

[ref33] Babu M.; Favretto F.; Ibáñez de Opakua A.; Rankovic M.; Becker S.; Zweckstetter M. Proline/arginine dipeptide repeat polymers derail protein folding in amyotrophic lateral sclerosis. Nat. Commun. 2021, 12 (1), 3396 10.1038/s41467-021-23691-y. 34099711 PMC8184751

[ref34] Camilloni C.; Sahakyan A. B.; Holliday M. J.; Isern N. G.; Zhang F.; Eisenmesser E. Z.; Vendruscolo M. Cyclophilin A catalyzes proline isomerization by an electrostatic handle mechanism. Proc. Natl. Acad. Sci. U. S. A. 2014, 111 (28), 10203–8. 10.1073/pnas.1404220111. 24982184 PMC4104850

[ref35] Zydowsky L. D.; Etzkorn F. A.; Chang H. Y.; Ferguson S. B.; Stolz L. A.; Ho S. I.; Walsh C. T. Active site mutants of human cyclophilin A separate peptidyl-prolyl isomerase activity from cyclosporin A binding and calcineurin inhibition. Protein Sci. 1992, 1 (9), 1092–9. 10.1002/pro.5560010903. 1338979 PMC2142182

[ref36] Ukmar-Godec T.; Hutten S.; Grieshop M. P.; Rezaei-Ghaleh N.; Cima-Omori M. S.; Biernat J.; Mandelkow E.; Soding J.; Dormann D.; Zweckstetter M. Lysine/RNA-interactions drive and regulate biomolecular condensation. Nat. Commun. 2019, 10 (1), 2909 10.1038/s41467-019-10792-y. 31266957 PMC6606616

[ref37] Jeener J. M. B. H.; Meier B. H.; Bachmann P.; Ernst R. R. Investigation of exchange processes by two-dimensional NMR spectroscopy. J. Chem. Phys. 1979, 71 (11), 4546–4553. 10.1063/1.438208.

[ref38] Dugave C.; Demange L. Cis-trans isomerization of organic molecules and biomolecules: implications and applications. Chem. Rev. 2003, 103 (7), 2475–532. 10.1021/cr0104375. 12848578

[ref39] Zhang X.; Vigers M.; McCarty J.; Rauch J. N.; Fredrickson G. H.; Wilson M. Z.; Shea J. E.; Han S.; Kosik K. S. The proline-rich domain promotes Tau liquid-liquid phase separation in cells. J. Cell Biol. 2020, 219 (11), e202006054 10.1083/jcb.202006054. 32997736 PMC7594490

[ref40] Wang P.; Heitman J. The cyclophilins. Genome Biol. 2005, 6 (7), 226 10.1186/gb-2005-6-7-226. 15998457 PMC1175980

[ref41] Gu J.; Liu Z.; Zhang S.; Li Y.; Xia W.; Wang C.; Xiang H.; Liu Z.; Tan L.; Fang Y.; Liu C.; Li D. Hsp40 proteins phase separate to chaperone the assembly and maintenance of membraneless organelles. Proc. Natl. Acad. Sci. U. S. A. 2020, 117 (49), 31123–31133. 10.1073/pnas.2002437117. 33229560 PMC7733851

[ref42] Darling A. L.; Dahrendorff J.; Creodore S. G.; Dickey C. A.; Blair L. J.; Uversky V. N. Small heat shock protein 22 kDa can modulate the aggregation and liquid-liquid phase separation behavior of tau. Protein Sci. 2021, 30 (7), 1350–1359. 10.1002/pro.4060. 33686711 PMC8197419

[ref43] Patel A.; Lee H. O.; Jawerth L.; Maharana S.; Jahnel M.; Hein M. Y.; Stoynov S.; Mahamid J.; Saha S.; Franzmann T. M.; Pozniakovski A.; Poser I.; Maghelli N.; Royer L. A.; Weigert M.; Myers E. W.; Grill S.; Drechsel D.; Hyman A. A.; Alberti S. A Liquid-to-Solid Phase Transition of the ALS Protein FUS Accelerated by Disease Mutation. Cell 2015, 162 (5), 1066–77. 10.1016/j.cell.2015.07.047. 26317470

