# Supplementary material for: Peptidyl Prolyl Isomerase A Modulates the Liquid–Liquid Phase Separation of Proline-Rich IDPs
Source: J Am Chem Soc. 2022 Aug 26;144(35):16157–63. doi: 10.1021/jacs.2c07149 (PMC9460772; doi:10.1021/jacs.2c07149)
Supplement: Supplementary file 1 — ja2c07149_si_001.pdf [file ja2c07149_si_001.pdf]

Supplementary Information for

Peptidyl Prolyl Isomerase A Modulates the Liquid-Liquid Phase Separation of Proline-rich IDPs.

Maria Babu[1], Filippo Favretto[1], Marija Rankovic[2] and Markus Zweckstetter[1, 2*]

[1]Deutsches Zentrum für Neurodegenerative Erkrankungen (DZNE), 37077 Göttingen; [2]Max Planck Institute for Multidisciplinary Sciences, Am Fassberg 11, D-37077 Göttingen, Germany.

Supplementary Methods

Protein and peptide preparation

PPIA was expressed and purified as described previously[1]. The mutant protein PPIA(R55A) was generated through site-directed mutagenesis using a QuikChange kit (Qiagen). PPIA(R55A) was expressed and purified in the same way as the wild-type protein. Tau protein was expressed and purified as described previously[2].

N-terminal acetylated and C-terminal amidated PR20 was produced by solid-phase peptide synthesis. Peptide stocks were prepared by weighing and dissolving the required amount of powder in the buffers used for LLPS assays and NMR spectroscopy as specified below.

PR20/tRNA LLPS

Yeast tRNA, Alexa-fluor 488™ (green) and Syto 17 were purchased from ThermoFisher Scientific, Invitrogen. RNAsin was purchased from Promega. TMR-labelled PR20 was purchased from GeneScript.

RNA-induced PR20 LLPS was performed according to[3]. To this end, PR20 in 25 mM HEPES buffer, containing 0.01% NaN_3, at pH 7.4, was mixed with yeast tRNA reaching final concentrations of PR20 and tRNA of 100 µM and 0.2 mg/ml, respectively. The assay was prepared in nuclease-free water and additionally contained 0.4 units/µl of RNAsin to prevent nuclease activity.

To visualize enrichment of PR20 inside droplets, the primary amine at the amidated C-terminus of PR20 was labelled with Alexa 488 following the micro-labelling kit instructions from ThermoFisher Scientific, Invitrogen. Excess dye after labelling was removed by dialysis. 2 µM of Alexa 488-labelled PR20 was then added to non-fluorescent PR20 solution. To demonstrate enrichment of RNA inside the droplets, Syto 17 RNA dye was added to the assay just before tRNA such that its final concentration was 100 µM. Micrographs were acquired at 63X magnification using the L5(green) and Y5(red) filters on Leica DM6B fluorescent microscope. Contrast was enhanced using Image J in Fig. 1 a for visualization purpose.

To characterize the recruitment of PPIA to PR20/tRNA droplets, droplets of PR20 with tRNA were prepared at 25°C such that their final concentrations, including the volume of added PPIA, was 100 µM and 0.2 mg/ml, respectively. To prevent dimerization of PPIA, 1 mM DTT was present. To the preformed droplets, PPIA was added at PR20: PPIA molar ratios of 1:0.05, 1:0.2 and 1:0.4. To each sample, 5 µM Alexa 488-labelled PPIA mixed with its unlabelled kind

to make up the required concentration, was also added. The same experiments were performed with the mutant PPIA(R55A) protein under identical conditions. To minimize the dilution effect on the droplets upon addition of PPIA or PPIA(R55A), their stocks in 25 mM HEPES buffer, 50 mM NaCl, 0.01 % NaN3, 0.5 mM DTT (pH 7.4) were concentrated and readjusted such that the added volume was only 4 % of the total assay volume in all cases. The samples were then observed under the microscope through the green fluorescence channel after 15 minutes of incubation at 25 °C. For calculation of recruitment ratios, raw green fluorescence images were used. The average intensity inside the droplet area was divided by the average intensity of a region of interest (ROI) just outside the droplet. Approximately 30 droplets of similar size were analysed for each condition. To compute the statistical significance of the difference between the PPIA and PPIA(R55A) data set for a specific molar ratio, an unpaired t-test was performed using Graph-Pad Prism. For visualization purpose, the brightness and contrast are enhanced uniformly in all images.

To test the effect of higher concentrations of PPIA on PR20/tRNA droplets, droplets of PR20 with tRNA were prepared at 25 °C such that their final concentrations in the assay after the addition of PPIA were 100 μM and 0.2 mg/ml, respectively. Alexa 488-labelled PR20 and DTT were present at final concentrations of 2 μM and 1 mM, respectively. Wild-type PPIA or PPIA(R55A) were added to the preformed droplets at PR20: PPIA molar ratios of 1:05, 1:1, 1:3, 1:5. Prior to its addition to PR20/tRNA droplets, PPIA or PPIA(R55A) (in 25 mM HEPES, 50 mM NaCl, 0.5 mM DTT and 0.01 % NaN_3, pH 7.4) were concentrated and readjusted such that the added volume constituted only 17 % of the total assay volume. To ensure that the dilution effect did not affect the droplets, an additional control experiment was performed, where just buffer without PPIA, corresponding to 17 % of total assay volume, was added to the preformed droplets. All samples were incubated for 15 minutes at 25° C prior to imaging with the microscope. For visualization purposes, images in Figure 1c were normalized to the same average background intensity before adjusting their contrast uniformly.

Granular areas were determined using ImageJ. To this end, the threshold value of intensity was set manually to pick up the area occupied by the fluorescently active droplets. The circularity value was set to 1. 'Analyse→Measure' (after selecting 'Area' in the 'Set Measurement' option) gave the area occupied by the droplets in the image. For all conditions, three to four images from different regions of the phase-separated sample were quantified.

Tau LLPS

Tau phase separation was achieved at 20 μM concentration of Tau in 25 mM HEPES buffer containing 1 mM TCEP at pH 7,4, 25 °C. To visualize the enrichment of Tau inside the droplets, the phase separation assay was spiked with 0.5 μM of Alexa-488 labelled Tau. Alexa 488 labelling of Tau was carried out according to the micro-labelling kit instructions from Thermofischer Scientific. Micrographs were acquired at 63X magnification using the L5(green) on Leica DM6B fluorescent microscope.

To estimate the recruitment of PPIA into Tau droplets, the Tau LLPS assay was prepared as described above, but without labelled Tau. To the preformed droplets, PPIA or PPIA(R55A) was added at Tau: PPIA/PPIA(R55A) molar ratios of 1:0.1 and 1:0.25. To each sample, 2.5 μM Alexa 488-labelled PPIA mixed with its unlabelled kind to make up the required concentration, was added. The volume of PPIA added at the end is limited to 13 % of the total volume so as avoid any dilution effect. The samples were then observed under the microscope after five minutes of incubation at 25 °C. The recruitment ratio was calculated as described for PR20. Approximately 20 droplets of similar size from two independent experiments were analysed for each condition. To compute the statistical significance of the difference between the PPIA and PPIA(R55A) data set for a specific molar ratio, an unpaired t-test was performed using Graph-Pad Prism.

The impact of PPIA or PPIA(R55A) on Tau droplets at higher concentrations was tested for Tau:PPIA/PPIA(R55A) molar ratios of 1:1, 1:2.5 and 1:5. Tau droplets were prepared as described above and spiked with 0.5 μM Alexa 488 labelled Tau. To the preformed droplets of Tau, PPIA or PPIA(R55A) was added. The volume of the PPIA added at the end was limited to 20 % of the total assay volume. A control experiment was also performed were buffer was added instead of PPIA to the preformed Tau droplets. Samples were observed under the microscope after five minutes of incubation. The granular area from the microscopic images was determined using ImageJ as described for PR20/tRNA LLPS experiments. Approximately eight images were quantified from each independent experiments and three independent experiments were performed per condition.

In order to calculate the fraction of PPIA-bound Tau inside the droplets, we used the fluorescence micrographs recorded for Tau droplets formed by 20 μM Tau in 25 mM HEPES buffer, 1 mM TCEP at pH 7.4, spiked with Alexa 488-labeled Tau. Because the fluorescence intensity outside the droplets was very low (e.g. Fig. 3c), we made the simplifying assumption

that Tau has been fully recruited into the droplet. Next, the average percentage area occupied by the droplet in the micrograph was estimated. This percentage area was extrapolated to the volume fraction occupied by the condensate assuming that the third dimension in the slice of the microscope focus is negligible. On the basis of these simplifications, we estimated the volume occupied by Tau droplets to be 1.5 % of the total volume. The concentration of Tau inside the droplets was then estimated based on the effective increase of the 20 μM Tau concentrated into 1.5 % sample volume, which results in a concentration of 1333 μM Tau inside the droplets. The concentration of PPIA (or PPIA(R55A)) inside the Tau droplets was estimated from an average recruitment ratio of 5.0 observed for the Tau:PPIA (or PPIA(R55A)) molar ratio of 1:0.25 and from 1.5 % volume occupied by the droplets. A similar recruitemnt ratio was assumed for the Tau:PPIA ratio of 1:0.5 and the concentration of PPIA inside droplets was estimated for that condition as well. On the basis of these estimated concentrations of Tau and PPIA inside the droplets, the following equation for a one-site binding model was used to estimate the fraction of PPIA-bound Tau inside the droplets:

$$K_d = \frac{P*L}{PL} \qquad (1)$$

where P is the concentration of free Tau, L is concentration of free PPIA and PL is the concentration of the Tau/PPIA complex inside the droplets. K_d is the binding affinity derived from the global fit analysis of the interaction of Tau with ^{15}N-labeled PPIA (or PPIA(R55A)).

Fluorescence recovery after photobleaching

FRAP experiments for PR20/tRNA droplets were performed on a Leica TCS SP8 confocal microscope with 63x oil immersion objective and a 561 DPSS laser line. For performing FRAP experiments, droplets of PR20/tRNA were spiked with 2 μM of TMR-labelled PR20**.** The region of interest (ROI) inside the droplet was bleached with fifteen iterations at 40 % laser power. The recovery was recorded over 500 frames, each corresponding to 523 ms. Experiments were performed on PR20/tRNA droplets between 15-35 minutes after its preparation, either only for PR20/tRNA droplets or for droplets in the presence of wild-type PPIA or PPIA(R55A) mutant protein. The PR20:PPIA/ PPIA(R55A) molar ratio was 1:0.4.

FRAP experiments for Tau protein droplets were performed on Zeiss LSM 880. Tau phase separation was achieved at 50 μM concentration of Tau in 100 mM PIPES buffer containing 1 mM MgSO4, 1 mM EGTA and 1mM DTT at pH 6,9 at 25 °C in the presence of 10 percent dextran. Droplets were spiked with Alexa 488 labelled Tau. The region of interest

inside the droplet inside the droplet was bleached with sixty iterations at 90 % laser power. The recovery was recorded over 424 cycles, each corresponding to 500 ms. Experiments were performed on Tau droplets soon after its preparation, either only for Tau droplets or for droplets in the presence of wild-type PPIA or PPIA(R55A) mutant protein. The Tau:PPIA/ PPIA(R55A) molar ratio was 1:0.5.

Images were analyzed using ImageJ. Fluorescence recovery was calculated for each time frame according to:

$$FRAP = \frac{I_{bleached} - I_{background}}{I_{av.prebleached}} \tag{2}$$

where $I_{bleached}$ is the intensity of the bleached region, $I_{background}$ is the intensity of the background and $I_{av.preblaeched}$ is the average intensity of the ROI over 5 frames before bleaching it.

The recovery curve obtained according to the above equation was then multiplied with an acquisition bleaching correction factor (ABCF) calculated as

$$ABCF = \frac{I_{reference} - I_{background}}{I_{av.reference}} \tag{3}$$

where $I_{av.\ reference}$ is the average intensity of an ROI that is not bleached within the droplet. The fluorescence recovery curve thus obtained was then normalized for representation. For each condition of PR20/tRNA droplets, fluorescence recovery of seven droplets of comparable size from two different samples were averaged. For each condition of Tau droplets, fluorescence recovery of three droplets of comparable size from two different samples were averaged.

NMR spectroscopy

^{15}N-labelled wild-type PPIA or the PPIA(R55A) mutant was titrated with PR20 in 25 mM HEPES buffer, 100 mM NaCl, 2 mM DTT, 0.01% NaN_3 and 10 % D20 at pH 7.4. Titrations were performed at 298K on a 800 MHz NMR spectrometer (Bruker) equipped with a cryoprobe. The concentration of the protein was fixed at 40 µM. ^{1}H-^{15}N HSQC spectra of the protein were recorded in the absence of PR20 and with PR20 at 9.6, 32, 96, 160 and 320 µM

concentrations. The spectra were processed with Topspin 3.6.2 (Bruker) and analysed with CCPN 2.4.2[4]

The dissociation constant, K_d, was calculated according to

$$1 - \frac{I}{I_0} = I_{max}\left[\frac{(K_d + P + x) - \sqrt{(K_d + P + x)^2 - 4 * P * x}}{2P}\right] \tag{4}$$

where I is the PPIA peak intensity in the presence of PR20, I_0 is the peak intensity of PPIA without PR20, P is the concentration of PPIA and x is the concentration of PR20.

The error in 1-(I/I_0) was calculated according to

$$\sigma_{(I/I_0)} = \left(\frac{I}{I_0}\right)\sqrt{\left(\frac{\sigma_{(I_0)}}{I_0}\right)^2 + \left(\frac{\sigma_{(I)}}{I}\right)^2} \tag{5}$$

where $\sigma_{(I/Io)}$ represents the error in I/I_0, $\sigma_{(I)}$ and $\sigma_{(Io)}$ are the RMS value of background noise in the HSQC spectrum of the protein in the presence and absence of PR20, respectively. The error value in K_d is the standard error of fitting.

^{15}N-labelled Tau was titrated with wild-type PPIA or mutant PPIA(R55A) in 50 mM sodium phosphate buffer, 1 mM TCEP and 10 % D20 at pH 6.8. Titrations were performed at 278 K on a 800 MHz NMR spectrometer (Bruker) equipped with a cryoprobe. ^{1}H-^{15}N HSQC spectra of 20 Tau µM were recorded in the absence and presence of 200 µM PPIA/PPIA(R55A). The spectra were processed with Topspin 3.6.2 (Bruker) and analysed with CCPN 2.4.2[4]

^{15}N-labelled wild type PPIA was titrated with Tau in 25 mM HEPES buffer, 100 mM NaCl, 1 mM TCEP and 10 % D20 at pH 7.4. . Titrations were performed at 298K on a 800 MHz NMR spectrometer (Bruker) equipped with a cryoprobe. The concentration of the protein was fixed at 18.4 µM. ^{1}H-^{15}N HSQC spectra of the protein were recorded in the absence of Tau and with Tau at 1.95, 4, 7.82, 16.3 and 32 fold excess concentrations with respect to PPIA. Similarly, HSQC spectrum of ^{15}N-labelled mutant PPIA(R55A) was recorded at the PPIA(R55A) concentration of 12 µM, in the absence and in the presence of Tau at PPIA: Tau ratio of 1:4, 1:6, 1:12 and 1:3.8. The spectra were processed with Topspin 3.6.2 (Bruker) and

analysed with CCPN 2.4.2[4]. The Kd value for PPIA-Tau intercation was estimated from the chemical shift perturbations according to the following equation,

$$CSP = CSP_{max}\left[\frac{(K_d + P + x) - \sqrt{(K_d + P + x)^2 - 4 * P * x)}}{2P}\right] \quad (6)$$

where

$$CSP = \sqrt{(\delta_H)^2 + (\frac{\delta_N}{6.5})^2}\,. \quad (7)$$

P is the concentration of PPIA and x is the concentration of Tau. The error value in K_d is the standard error of fitting.

Quantification of PPIA-enhanced isomerization

A two-dimensional NOESY spectrum of 100 µM PR20 was recorded on a 800 MHz NMR spectrometer (Bruker) at 298 K in 100 mM K_2HPO_4/KH_2PO_4 buffer, 10% D_2O, pH 7 using a NOE mixing time of 300 ms. The spectrum was assigned with the help of a two-dimensional TOCSY spectrum (mixing time of 80 ms) recorded for the same sample.

To estimate the rate of cis/trans-exchange of prolines in PR20 in the presence of PPIA, NOESY spectra were recorded for mixing times ranging from 50 ms to 400 ms for the following PPIA:PR20 molar ratios:

PPIA:PR20	PR20 conc.(µM)	Spectrometer
1:30	300	900 MHz
1:8	100	800 MHz
1:4	100	800 MHz
1:1.5	100	800 MHz

The buffer and temperature conditions were identical to the reference condition. The spectra were processed with Topspin 3.6.2 (Bruker) and analysed with CCPN 2.4.2[4]

k_{ex} values, defined as sum of back and forward rates of a two-state model, were obtained according to[5]

$$\frac{I_{ex}}{I_{trans}} = \frac{1 - e^{(-k_{ex})*MT}}{e^{-k_{ex}*MT} + \frac{1 - X_{cis}}{X_{cis}}} \tag{8}$$

where I_{ex} is the intensity of the exchange peak between the cis and trans isoforms of H^{δ} of prolines, I_{trans} is the intensity of the of H^{δ} of the trans conformation, MT is the mixing time and X_{cis} is the fraction of the cis population. H^{δ} of prolines were chosen for analysis due to the well separated cis and trans peaks of these protons. The T_1 relaxation is considered to be similar for cis and trans proline peptides. Experimental data were fitted against the given equation, keeping k_{ex} and X_{cis} as free fit parameters, using least square fitting. The k_{ex} values were determined from both the exchange peaks on either side of the diagonal, and the k_{ex} reported is the average of the two k_{ex} values derived per condition. The error in k_{ex} is the standard deviation from the average value.

Additionally, a similar analysis was done with the intensity ratio of the exchange peak (I_{ex}) to the cis diagonal peak (I_{cis}). In this case, the equation 8 was modified as follows

$$\frac{I_{ex}}{I_{cis}} = \frac{1 - e^{(-k_{ex})*MT}}{e^{-k_{ex}*MT} + \frac{1 - X_{trans}}{X_{trans}}} \tag{9}$$

where I_{ex} is the intensity of the exchange peak between the cis and trans isoforms of H^{δ} of prolines, I_{cis} is the intensity of the of H^{δ} of the cis conformation, MT is the mixing time and X_{trans} is the fraction of the trans population. X_{trans} was kept as free fit parameter during fitting. The k_{ex} were determined from both the exchange peaks on either side of the diagonal, and the k_{ex} reported is the average of the two kex values derived per condition. The error in k_{ex} is the standard deviation from the average value.

For determination of k_{ex} for PR20 in the presence of PPIA(R55A), the experiments were performed as described above in 100 mM K_2HPO_4/KH_2PO_4 buffer, 10% D_2O at pH 7.4 on a 700 MHz NMR spectrometer equipped with a cryoprobe.

The error in (I_{ex}/I_{trans}) was calculated from the noise in the NOESY spectra according to:

$$\sigma_{(I_{ex}/I_{trans})} = \left(\frac{I_{ex}}{I_{trans}}\right)\sqrt{\left(\frac{\sigma_{(I_{ex})}}{I_{ex}}\right)^2 + \left(\frac{\sigma_{(I_{trans})}}{I_{trans}}\right)^2} \qquad (10)$$

where $\sigma_{(Iex/Itrans)}$ is the error in I_{ex}/I_{trans}, $\sigma_{(Iex)}$ and $\sigma_{(Itrans)}$ are the noise (RMS value of background noise) in the NOESY spectrum.

The two-dimensional ROESY spectrum of PR20/PPIA was recorded with 400 µM PR20 and a PR20:PPIA molar ratio of 8:1 on a 800 MHz NMR spectrometer (Bruker) using the same buffer and temperature conditions as used for the NOESY experiments. The mixing time for the ROESY experiment was 220 ms.

Supplementary Figure 1

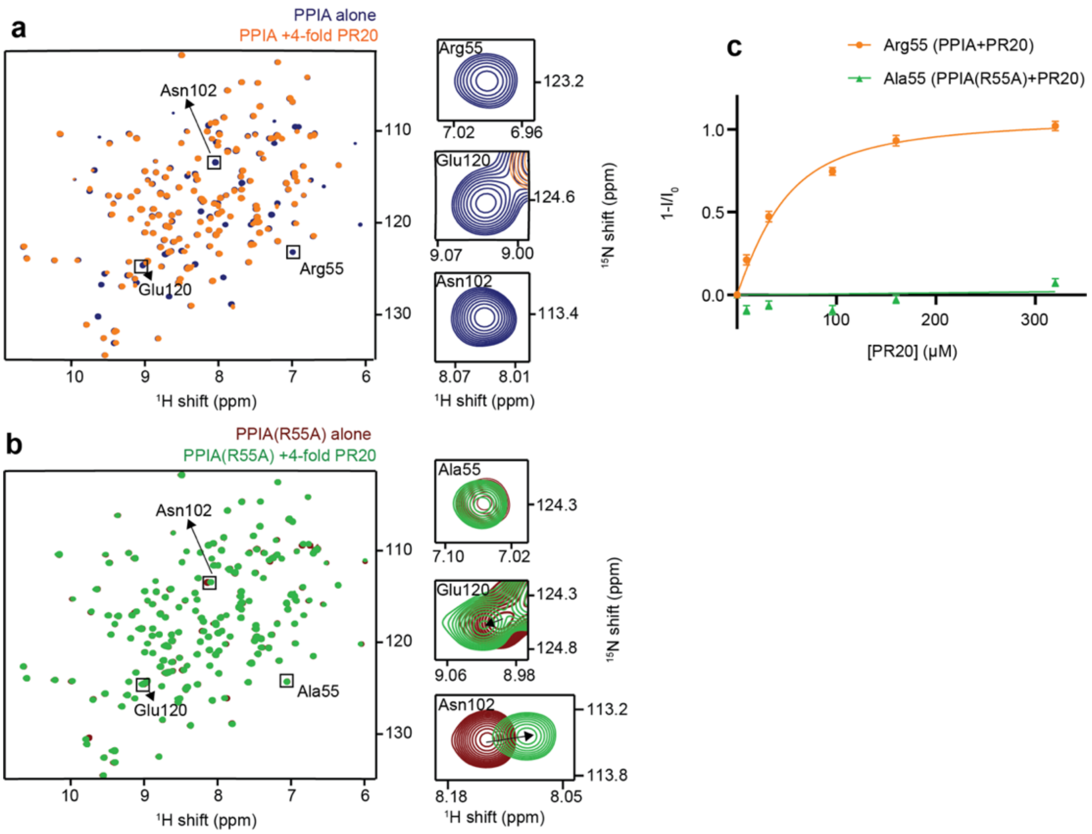

Figure S1. Binding of PR20 to wild-type and mutant PPIA. **a)** Two-dimensional ^{1}H-^{15}N HSQC spectra of PPIA alone (blue) and in presence of a 4-fold excess of PR20 (orange). Highlighted are the cross peaks of Arg55, Glu120 and Asn102, which are predominantly in the slow exchange interaction regime. These residues are in direct contact with PR20 in the PPIA/PR20 complex[1]. **b)** ^{1}H-^{15}N HSQC spectra of PPIA(R55A) alone (maroon) and in the presence of a 4-fold excess of PR20 (green). The cross peak of Ala55 is unaffected by the addition of PR20, Glu120 shows very small chemical shift changes while Asn102 is in fast-to-intermediate exchange. **c)** Intensity changes of the cross peak of Arg55 in PPIA and Ala55 in PPIA(R55A), as a function of increasing concentration of PR20. The lines represent least-square fitting of the experimental data of Arg55 from which the K_d value was derived. The K_d value for the PPIA/PR20 interaction derived from the Arg55 cross peak is 23± 7.28 μM. The same could not be estimated for the PPIA(R55A)/PR20 interaction because of minimal perturbations. Error bars represent error in $1-I/I_0$ calculated from the noise in the NMR spectra.

Supplementary Figure 2

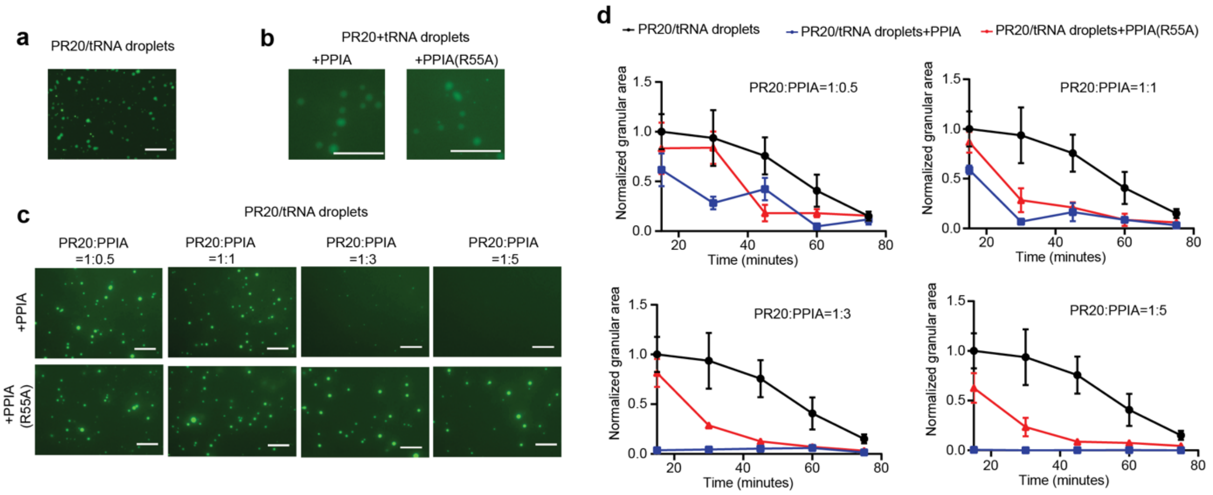

Figure S2. PPIA interferes with RNA-induced LLPS of PR20. **a)** LLPS of PR20 into PR20/tRNA droplets. Droplets of PR20/tRNA were obtained by mixing 100 µM PR20 with 0.2 mg/ml of tRNA. Droplets were visualized by addition of Alexa488-labelled PR20 (green). Images are taken after 15 minutes of incubation. Scale bar, 20 µm. **b)** Micrographs showing the recruitment of PPIA (left) and PPIA(R55A) (right) into PR20/tRNA droplets. Recruitment of PPIA variants into the preformed droplets are shown for the PR20:PPIA and PR20:PPIA(R55A) molar ratio of 1:0.2. Localization of PPIA variants inside the PR20/tRNA droplets were visualized by labelling them with Alexa-488 green fluorescent dye. Images were obtained after 15 minutes of incubation. Scale bar, 20 µm. **c)** PPIA-induced dissolution of PR20/tRNA droplets. Fluorescence images of Alexa488-labelled PR20/tRNA droplets at increasing PPIA (top) and PPIA(R55A) (bottom) concentrations are shown from left to right. Images are obtained after 15 minutes of incubation. Scale bar, 20 µm. **d)** Average granular area occupied by PR20/tRNA droplets after addition of wild-type PPIA (blue) or mutant PPIA(R55A) (red), followed in time. Average granular area in a control sample without any PPIA variant is displayed in black. Error bars represent the standard deviation from average area calculated from four different micrographs.

Supplementary Figure 3

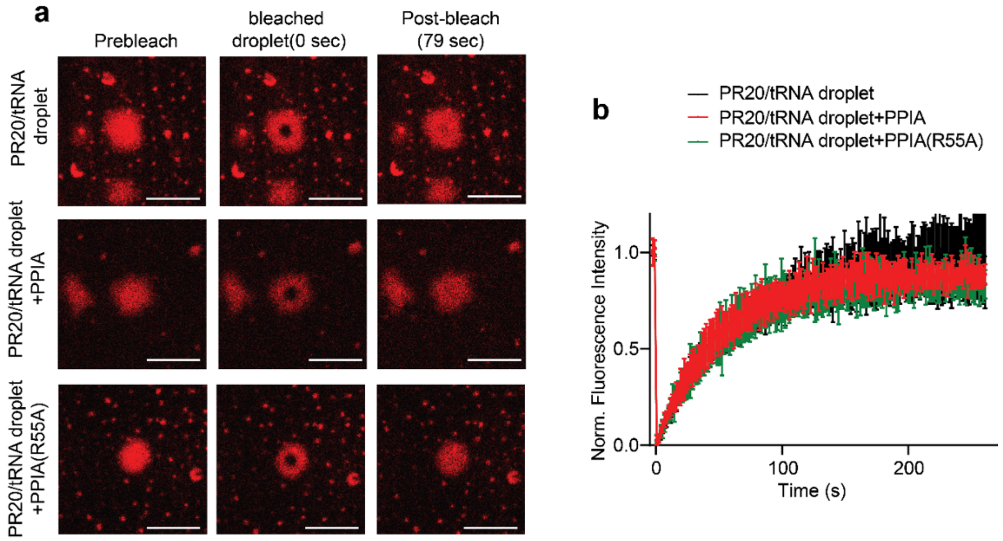

Figure S3. Fluorescence recovery of TMR-labelled PR20 inside PR20/tRNA droplets. **a)** Micrographs showing PR20/tRNA droplets (top row), PR20/tRNA droplets in the presence of PPIA (middle row) and in presence of PPIA(R55A) (bottom row) before bleaching, soon after bleaching (0 sec) and 79 seconds after bleaching. PPIA variants are added to preformed droplets of PR20/tRNA at PR20:PPIA variant ratio of 1:0.4. Scale bar, 5 µm. **b)** Averaged fluorescence recovery curve after photobleaching of PR20/tRNA droplets alone (black), and in the presence of PPIA (red) and PPIA(R55A) (green). Error bars represent standard deviation from average recovery curve calculated from seven droplets per condition.

Supplementary Figure 4

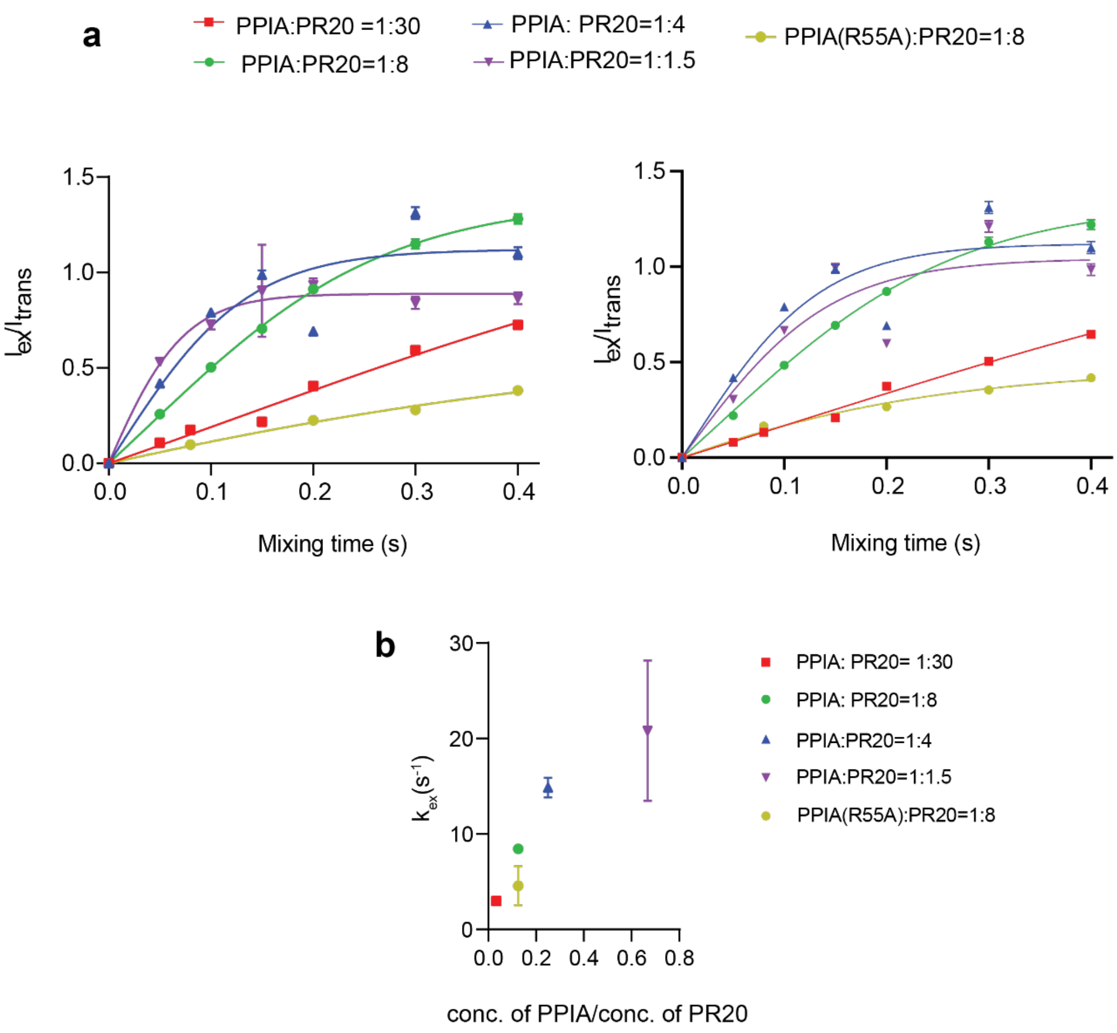

Figure S4. Isomerase activity of PPIA on the dipeptide repeat protein PR20. a) Ratios between the intensity of the cis/trans-exchange peak of proline H^{δ}, I(ex), and the intensity of its cis diagonal peak, I(cis), as a function of mixing time of the NOESY experiment for PPIA:PR20 molar ratios of 1:30 (red, square), 1:8 (green, circle),1:4 (blue, triangle) and 1:1.5 (magenta, inverted triangle), as well as for PPIA(R55A):PR20 molar ratio of 1:8 (yellow, circle). Lines represents least-square fittings of the data to obtain the exchange rate k_{ex}. Error bars represent error in I_{ex}/I_{cis} calculated from the noise in the NMR spectra. The graphs on left and right represent the same analysis but the I_{ex} value in the two cases are taken from the two exchange peaks on either side of diagonal, which are marked by rectangular boxes in Fig 2a (middle). b) Rates of cis/trans-interconversion, k_{ex}, in PR20 for different PPIA:PR20 ratios derived from fitting the I_{ex}/ I_{cis} value corresponding to various mixing times against equation 9. k_{ex} values here are the average of the two k_{ex} values, derived per condition, from the two different fitting analysis shown in panel a. Error bars represent standard deviations from the average k_{ex} value.

Supplementary Figure 5

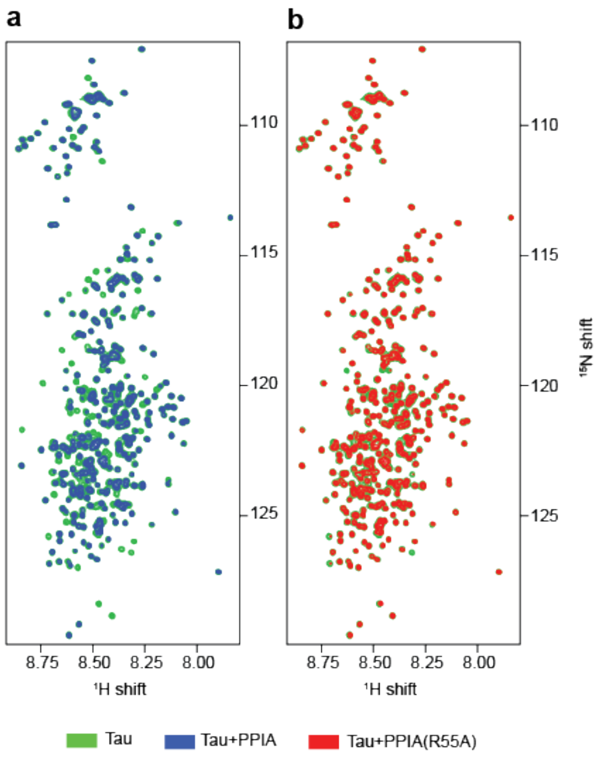

Figure S5. Binding of Tau to wild-type and mutant PPIA. **a)** Superposition of two-dimensional ^{1}H-^{15}N HSQC spectra of Tau alone (green) and in the presence of a 10-fold excess of PPIA (blue). **b)** Superposition of the ^{1}H-^{15}N HSQC spectra of Tau alone (green) and in the presence of a 10-fold excess of PPIA(R55A) (red).

Supplementary Figure 6

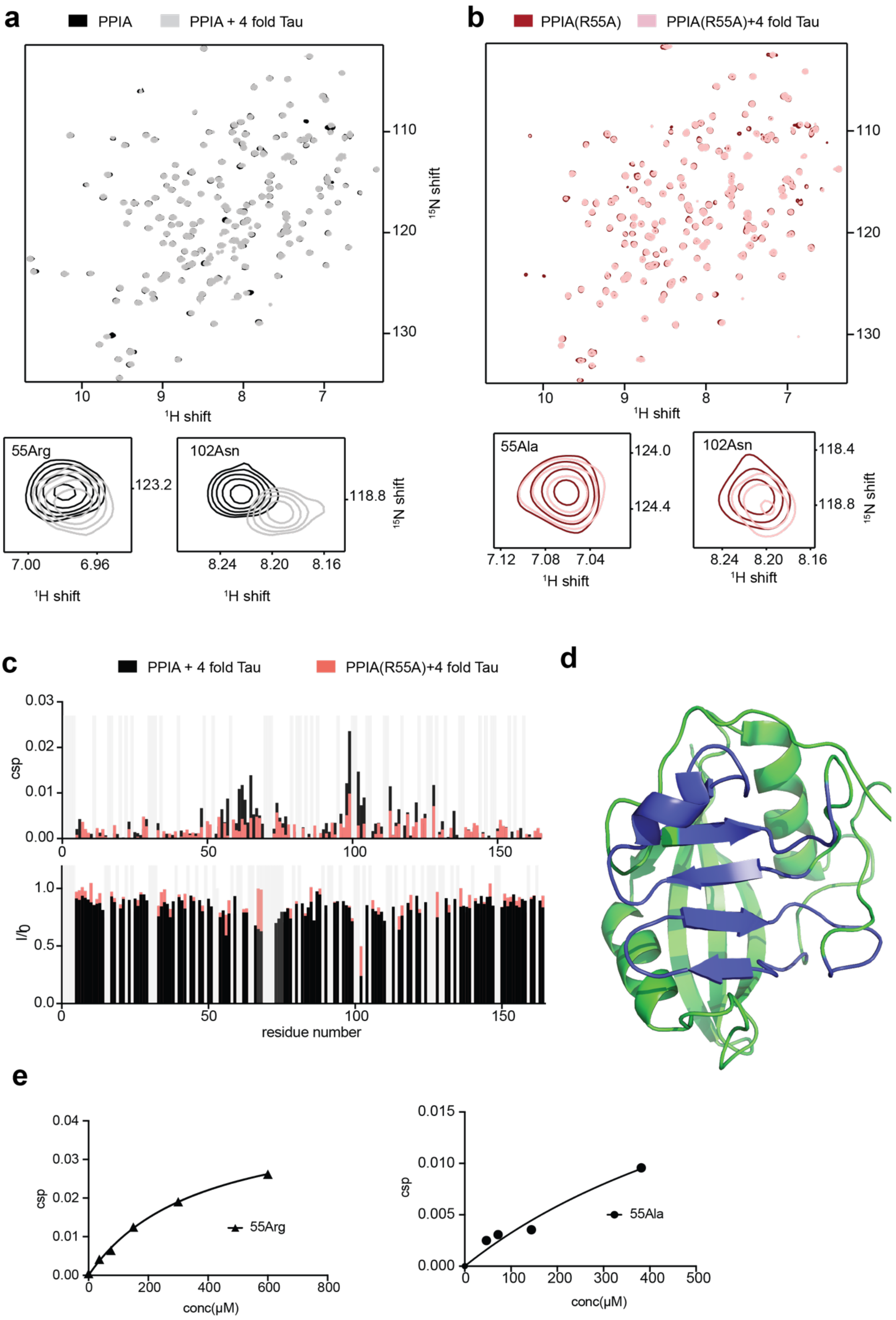
a
PPIA
PPIA + 4 fold Tau
110
120
130
15N shift
10
9
8
7
1H shift
55Arg
123.2
7.00
6.96
1H shift
102Asn
118.8
8.24
8.20
8.16
1H shift
b
PPIA(R55A)
PPIA(R55A)+4 fold Tau
110
120
130
15N shift
10
9
8
7
1H shift
55Ala
124.0
124.4
7.12
7.08
7.04
1H shift
102Asn
118.4
118.8
8.24
8.20
8.16
1H shift
c
PPIA + 4 fold Tau
PPIA(R55A)+4 fold Tau
0.03
0.02
0.01
0.00
csp
0
50
100
150
1.0
0.5
0.0
I/I0
residue number
d
e
0.04
0.03
0.02
0.01
0.00
csp
0
200
400
600
800
conc(μM)
55Arg
0.015
0.010
0.005
0.000
csp
0
100
200
300
400
500
conc(μM)
55Ala

Figure S6. Binding of Tau to wild-type and mutant PPIA. **a)** Two-dimensional ^1H-^{15}N HSQC spectra of PPIA alone (black) and in presence of a 4-fold excess of Tau (grey). Highlighted are the cross peaks of Arg55 and Asn102 that are present in the binding site of PPIA. **b)** ^1H-^{15}N HSQC spectra of PPIA(R55A) alone (maroon) and in the presence of a 4-fold excess of Tau (lightpink). **c)** Single-residue analysis of the interaction of Tau with wild-type and mutant PPIA. Chemical shift perturbations and intensity perturbations of ^1H-^{15}N HSQC peaks of PPIA (black) and PPIA(R55A) (pink) upon addition of 4-fold excess of Tau are shown above and below, respectively. Grey bars represent residues that were excluded from the analysis. **d)** Residues of PPIA that shows significant chemical shift perturbations due to interaction with Tau are mapped onto the crystal structure of PPIA (PDB code: 5kuz) in blue colour. **e)** Chemical shift perturbations of the cross peaks of Arg55 of PPIA (left), and of Ala55 of PPIA(R55A) (right), as a function of increasing concentration of Tau. The line represent least-square fitting of the experimental data. The K_d value for the PPIA/Tau interaction derived from the Arg55 cross peak is 353 ± 30 μM μM. The same for PPIA(R55A)/Tau interaction derived from Ala55 is 817 ± 74 μM.

Supplementary Figure 7

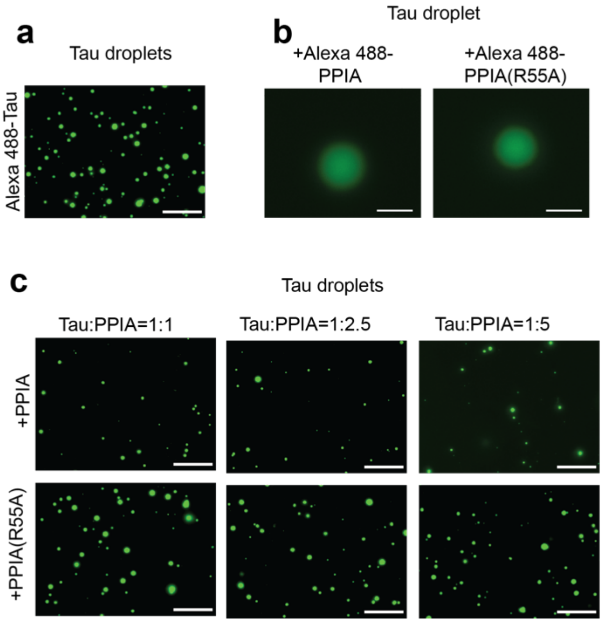

Figure S7. PPIA reverses Tau LLPS. **a)** Phase separation of Tau into droplets. LLPS was achieved at 20 µM Tau concentration in low ionic strength buffer condition. Droplets were visualized by addition of Alexa488-labelled Tau (green). Images were obtained after five minutes of incubation. Scale bar, 30 µm. **b)** Recruitment of PPIA (left) and PPIA(R55A) (right) to a Tau droplet. The Tau to PPIA or PPIA(R55A) molar ratio was 1:0.1. The PPIA variants were labelled green with the fluorescent dye Alexa-488 . Images were obtained after five minutes of incubation. Scale bar, 5 µm. **c)** PPIA-induced dissolution of Tau droplets. Fluorescence images of Alexa488-labelled Tau droplets at increasing PPIA (top) and PPIA(R55A) (bottom) concentrations are shown from left to right. Images were obtained after five minutes of incubation. Scale bar, 30 µm.

Supplementary Figure 8

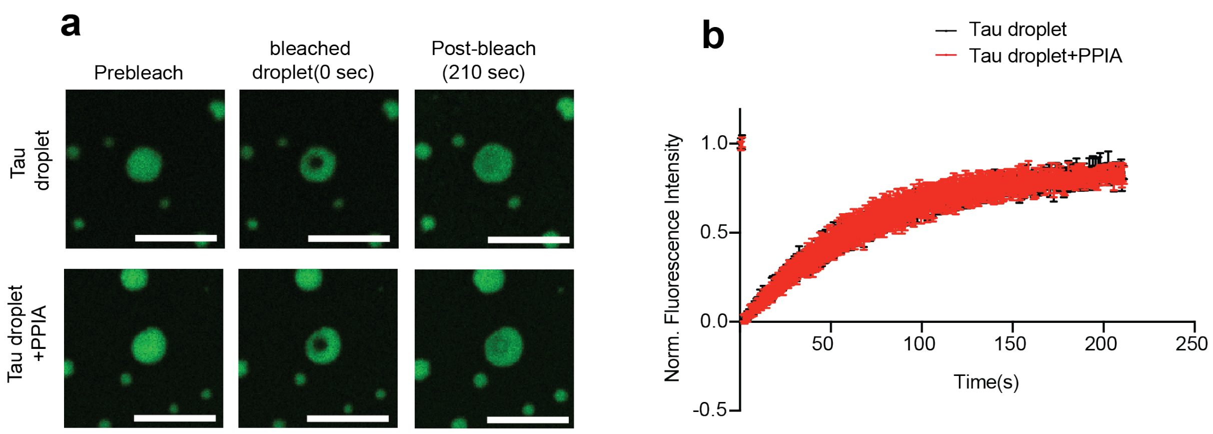

Figure S8. Fluorescence recovery of Alexa488-labelled Tau inside Tau droplets. **a)** Micrographs showing Tau droplets (top row), and Tau droplets in the presence of PPIA (bottom row) before bleaching, immediately after bleaching (0 sec) and 210 seconds after bleaching. PPIA variants were added to preformed droplets of Tau at PR20:PPIA variant ratio of 1:0.5. Scale bar, 6 µm. **b)** Averaged fluorescence recovery curve after photobleaching of Tau droplets alone (black), and in the presence of PPIA (red). Error bars represent standard deviation from average recovery curve calculated from three droplets per condition.

Supplementary References

1. Babu, M.; Favretto, F.; Ibáñez de Opakua, A.; Rankovic, M.; Becker, S.; Zweckstetter, M., Proline/arginine dipeptide repeat polymers derail protein folding in amyotrophic lateral sclerosis. *Nat Commun* **2021,** *12* (1), 3396.
2. Ukmar-Godec, T.; Hutten, S.; Grieshop, M. P.; Rezaei-Ghaleh, N.; Cima-Omori, M. S.; Biernat, J.; Mandelkow, E.; Soding, J.; Dormann, D.; Zweckstetter, M., Lysine/RNA-interactions drive and regulate biomolecular condensation. *Nat Commun* **2019,** *10* (1), 2909.
3. Boeynaems, S.; Bogaert, E.; Kovacs, D.; Konijnenberg, A.; Timmerman, E.; Volkov, A.; Guharoy, M.; De Decker, M.; Jaspers, T.; Ryan, V. H.; Janke, A. M.; Baatsen, P.; Vercruysse, T.; Kolaitis, R. M.; Daelemans, D.; Taylor, J. P.; Kedersha, N.; Anderson, P.; Impens, F.; Sobott, F.; Schymkowitz, J.; Rousseau, F.; Fawzi, N. L.; Robberecht, W.; Van Damme, P.; Tompa, P.; Van Den Bosch, L., Phase Separation of C9orf72 Dipeptide Repeats Perturbs Stress Granule Dynamics. *Mol Cell* **2017,** *65* (6), 1044-1055 e5.
4. Vranken, W. F.; Boucher, W.; Stevens, T. J.; Fogh, R. H.; Pajon, A.; Llinas, M.; Ulrich, E. L.; Markley, J. L.; Ionides, J.; Laue, E. D., The CCPN data model for NMR spectroscopy: development of a software pipeline. *Proteins* **2005,** *59* (4), 687-96.
5. Monneau, Y. R.; Soufari, H.; Nelson, C. J.; Mackereth, C. D., Structure and activity of the peptidyl-prolyl isomerase domain from the histone chaperone Fpr4 toward histone H3 proline isomerization. *J Biol Chem* **2013,** *288* (36), 25826-25837.
